# Prevalence of coronary artery calcification in a multiethnic population in Angola

**DOI:** 10.21542/gcsp.2023.12

**Published:** 2023-05-11

**Authors:** Humberto Morais, Preciosa Lourenço, Carlos Martins, Lorette Cardona, Mauer A. A. Gonçalves

**Affiliations:** 1 Centro de estudos Avançados em Educação e Formação Médica, Universidade Agostinho Neto; 2 Hospital Militar Principal/Instituto Superior, Luanda, Angola; 3 Faculdade de Medicina da Universidade Agostinho Neto, Luanda, Angola; 4 Luanda Medical Center, Luanda, Angola

## Abstract

Summary: This article aims to study the prevalence of coronary artery calcification and associated factors in a multiethnic population in Angola.

Methods: A descriptive, cross-sectional observational study was carried out in a private clinic in Angola. For this purpose, information was collected from sociodemographic and biological data. The selected variables were; history of arterial hypertension, diabetes mellitus, smoking (current and past), alcohol consumption, family history of coronary disease, and coronary calcium score. Independent Mann–Whitney test, Student’s *t*-test and chi-square test were used as appropriate.

Results: The sample consisted of 211 individuals: 156(73.9%) of black race, 37(17.4%) of mixed race and 18(8.4%) of Caucasian race. 126(59.7%) were male. The average age was 56.7 ± 9.3 years. Of the total sample, 158 (74.9%) had a history of hypertension, 50 (23.7%) of diabetes mellitus, and 138 (65.4%) of dyslipidemia. Of the total number of individuals, 21(10.0%) were smokers and 38(18.0%) were ex-smokers, 137 (64.9%) were social drinkers and 44(20.9%) were obese. A significant association was found between calcification of the coronary arteries and aging (p <.001), Caucasian race (p =.037), and a history of diabetes mellitus, dyslipidemia and smoking (p <.001, p <.001, p =.012, respectively). Black race and female gender are associated with a lower risk of coronary artery calcification (p =.034 and p =.011, respectively).

Conclusion: The present results support the notion that there are racial and ethnic differences in the prevalence of coronary calcification.

## Introduction

Several studies have documented ethnic and racial differences in the prevalence of coronary artery calcification (CAC)^1^. However, data are controversial^2–10^; the vast majority of studies indicate that coronary artery calcification is less prevalent in blacks than in whites^2–6^ although this finding is not universal^7–10^ In the study carried out by Orimoloye et al. where including 42,224 individuals, the prevalence of CAC was higher in blacks (56.1%), followed by whites (55.8%), Hispanics (54.0%) and Asians (51.0%)^9^.

The underlying cause(s) of racial differences in CAC are not understood. Differences in the frequency of cardiovascular disease (CVD) risk factors or the strength of their relationship with CAC may play a determining role^11^.

In Africa, data on the use of coronary CT are scarce^12^. In a study carried out by Adjaye et al. in Ghana on the coronary calcium score, the authors reported a prevalence of coronary artery calcification of 21.2%, the distribution of CAC was significantly affected by age, but there was not enough evidence to conclude on the effect of sex on the prevalence of CAC. It should be noted that this study included only black patients, therefore, it is not possible to compare ethnic differences in the prevalence of coronary calcium score in this population.

To the best of our knowledge, there have been no studies on this topic in Angola. The main objective of this study was to evaluate the prevalence of coronary artery calcification in a multiethnic population in Angola.The secondary objectives were (1) to compare the prevalence of CAC by sex, ethnicity/race, and its relationship with age, and (2) to identify predictive factors for the presence of CAC in our population.

## Methodology

### Type and place of study

A retrospective observational cohort study was conducted, including all users who underwent computed tomography angiography of the coronary arteries at the Clínica Luanda Medical Center between October 2019 and May 2022.

### Study population

The study population consisted of all users who underwent computed tomography angiography of the coronary arteries and had their data included in the database at Clínica Luanda Medical Center during the referred period. The 233 patients who underwent cardiac CT angiography were listed in the database. Patients were divided into three groups according to race/ethnicity: Group I - Black, Group II - Mixed, Group III – White.

The study was approved by the Directorate of Clínica Luanda Medical Center by the direction of CEDUMED. The preservation and confidentiality of patient information were guaranteed following all standards for research on human beings in accordance with the Declaration of Helsinki.

### Study variables

Demographic and clinical variables (cardiovascular risk factors), and coronary calcium score (CCS) were also included. The variables were initially collected from the database at the Clínica Luanda Medical Center and later from the patients’ clinical files, whenever necessary.

### Definition of risk factors

Hypertension was defined as a previous diagnosis of hypertension or treatment with antihypertensive therapy. Diabetes mellitus was defined as a previous diagnosis of diabetes mellitus or treatment with anti-diabetic drugs. Dyslipidemia was defined as a previous diagnosis of dyslipidemia or treatment with any lipid-lowering medication. Obesity was defined as having a BMI > 30 kg/m^2^. Smoking status was defined based on the current and past smoking status (yes/no). Alcoholic habits were defined based on the current status (yes/no). A family history of coronary artery disease (CAD) was determined by the presence of a first-degree relative with a history of CAD.

### Coronary arterial calcium calculation

Computed tomography for the calculation of CCS was performed using a 64-slice multidetector computed tomography scanner (Somatom Perspective; Siemens, Erlagen, Germany) with the following parameters: tube voltage 100–120 kV, collimation 64 mm ×0.6 mm and temporal resolution 0.185 s. The exams were performed with prospective electrocardiographic gating without contrast,

The evaluation score was calculated by Syngo.via Cardiac following the standard methodology described by Agatston et al.^13^. Acquisition was performed using 3-mm slices, followed by reconstruction to a slice thickness of 0.75 mm. Images were acquired mainly at the end of inspiration, and developed from the level of the carina to the base of the heart. Patients were divided into three groups according to their coronary calcium score (CCS): Group I –CCS = 0 Group II - CCS 1–100; Group III - CCS > 100. The scans were reported by a cardiologist with extensive experience in cardiac imaging.

### Inclusion and exclusion criteria

Consecutive patients aged 18 years or older who underwent a coronary calcium scan and whose sociodemographic and clinical data were collected and entered into the clinic’s database were included. Patients with a history of previous coronary revascularization, those who did not undergo a coronary calcium scan, and those who underwent cardiac CT angiography for indications other than suspected coronary artery disease were excluded.

### Statistical analysis

The normality of the distribution was analyzed using the Kolmogorov–Smirnov test. Qualitative variables were expressed as absolute and relative frequencies and quantitative variables as mean ± standard deviation (SD). Independent Mann–Whitney test, Student’s *t*-test and chi-square test were used as appropriate. Statistical significance was defined as p <0.05. The analysis was performed using the Statistical Package for the Social Sciences program (SPSS, version 21.0).

## Results

A total of 211 patients were included in the study. The mean age was 56.72 ± 9.31. 126 (59.7%) of the patients were male. 156 (73.9%) were black, 37 (17.5%) were mixed race, and 28 (8.5%) were white. In 137 (64.9%) patients, the CCS was equal to zero, 43 (20.4%) patients had a CCS between 1 and 100 and 31 (14.7%) had a CCS>100.

Demographic and clinical characteristics and CCS findings in the total population according to sex are presented in Table 1. There were no significant differences in age or race/ethnicity between men and women. Regarding hypertension, obesity, dyslipidemia, and family history of CAD, there were no differences between sexes. On the other hand, in relation to men, women had less diabetes mellitus (p =.043), fewer smoking habits in the past (p <.002), and less alcohol consumption (p <.001). The CCS was also significantly lower in women than in men (p =.011).

**Table 1:** Demographic, clinical characteristics and findings of coronary CT angiography in the total population and according to gender.

	Total (n = 211)	Male (n = 126)	Female (n = 85)	p-Value
Age	56.72 ± 9,31	56,80 ± 9,46	56,60 ± 9,13	.878
Race/Ethnicity				.172
Black, n (%)	156 (73,9)	93 (73,8)	63 (74,1)	
Mixed, n (%)	37 (17,5)	19 (15,1)	18 (21,2)	
Caucasian, n (%)	18 (8,5)	14 (11,1)	4 (4,7)	
Risk factors				
Arterial hypertension, n (%)	158 (74,9)	93 (73,8)	65 (76,5)	.662
Diabetes mellitus, n (%)	50 (23,7)	36 (28,6)	14 (16,5)	.043^*^
Dyslipidemia, n (%)	138 (65,4)	88 (69,8)	50 (58,8)	.099
Smoking, n (%)	21 (10,0)	13 (10,3)	8 (9,4)	.829
Former tabagism, n	38 (18,0)	31 (24,6)	7 (8,2)	.002^**^
Obesity, n (%)	44 (20,9)	23 (18,3)	21 (24,7)	.258
Alcoholic habits, n (%)	137 (64,9)	99 (78,6)	38 (44,7)	<.001^***^
FH of CHD n (%)	17 (8.1)	10 (7,9)	7 (8,2)	.938
Known CHD, n (%)	4 (1,9)	4 (3,2)	0 (0)	.097
Coronary calcium score				.011^*^
CCS 0 n (%)	13 (64,9)	72 (57,1)	65 (76,4)	
CCS 1-100 n (%)	43 (20,4)	33 (26,2)	10 (11,8)	
CCS >100 n (%)	31 (14,7)	21 (16,7)	10 (11,8)	
Percentiles of CCS				
Median	0	0	0	–
25th percentile	0	0	0	–
75th percentile	29.00	51.00	0	–
90th percentile	226.20	279.10	153.4	–
95th percentile	432.20	545.00	335.5	–
Maximum	2088.00	2088.00	1586.0	–

**Notes.**

CHD Coronary heart disease CSS Coronary calcium score FH Family history

* p<.05.

** p<.01.

*** p<.001.

Demographic data, clinical characteristics, and coronary calcium score findings in the total population and according to ethnicity, are presented in Table 2. There were no significant differences in age and sex between ethnicities. Compared to black patients, mixed-race and Caucasian patients had a higher percentage of dyslipidemia (p =0.060), smoking (p <.001) and alcohol consumption (p =.013). Furthermore, Caucasians had a significantly higher prevalence of a family history of CAD than the other two groups (p <.003). CAC was significantly higher in Caucasians than in Black and mixed-race patients (p .037). There was no significant difference between black and mixed-race individuals with their CAC scores.

**Table 2:** Distribution of demographics, cardiovascular risk factors and coronary calcium score in the total population and according to race/ethnicity.

	Total (n = 211)	Black (n = 156)	Mixed (n = 37)	Caucasian (n = 18)	p-Value
Age	56.72 ± 9,31	56.21 ± 9.53	58.24 ± 9.27	58.00 ± 7.13	.409
Gender					.172
Male n (%)	126 (59,7)	93 (59.6)	19 (51,4)	14 (77.8)	
Female n (%)	85 (40,3)	63 (40.4)	18 (48.6)	4 (22.2)	
Risk factors					
Arterial hypertension, n (%)	158 (74,9)	121 (77,1)	26 (70,3)	11 (61,1)	.273
Diabetes mellitus, n (%)	50 (23,7)	35 (22,3)	10 (27,0)	5 (27,7)	.754
Dyslipidemia, n (%)	138 (65,4)	95 (60,5)	29 (78,4)	14 (77,7)	.060
Smoking, n (%)	21 (10,0)	9 (5,7)a	5 (13,5)	7 (38,9)	<.001^***^
Former tabagism, n	38 (18,0)	22 (14,5)	12 (32,4)	4 (22,2)	.027^*^
Obesity, n (%)	44 (20,9)	28 (17,8)	12 (32,4)	4 (22,2)	.141
Alcoholic habits, n (%)	137 (64,9)	93 (59,2)	28 (75,8)	16 (88,8)	.013^*^
FH of CHD n (%)	17 (8.1)	11 (7.0)	1 (2,7)	5 (27,8)	.003^**^
Prevalence of CAC n (%)	4 (1,9)	48(30.4)	16(43.2)	11(61.1)	.018^*^
Coronary Calcium Score					.037^*^
CCS 0 n (%)	137 (64,9)	109 (69.9)	21(56.8)	7 (38.9)	
CCS 1–100 n (%)	43 (20,4)	29 (18.6)	7 (18.9)	7 (38.9)	
CCS>100 n (%)	31 (14,7)	18 (11.5)	9 (24.3)	4 (22.2)	
Percentiles of CCS					
Median	0	0	0	0	–
25th percentile	0	0	0	8,5	–
75th percentile	29.00	14,5	133.0	88.5	–
90th percentile	226.2	154.1	621.8	411.4	–
95th percentile	432.2	349.5	889.1	595.0	–
Maximum	2088.0	2088	1187	595	–

**Notes.**

CAC Coronary artery calcification CHD Coronary heart disease CSS Coronary calcium score FH Family history

* p<.05.

** p<.01.

*** p<.001.

The demographic and clinical characteristics and findings of the CCS in the total population are presented in Table 3. Regarding age, a significant difference was observed among the three groups (p <.001), reflecting an increase in CCS with aging. We also found a significantly higher percentage of men in the groups with CCS between 1–100 and > 100 when compared to the group where the CCS was 0 (zero) (p. 0.011). The prevalence of diabetes mellitus, dyslipidemia, and ex-smokers was also significantly higher in patients with CCS between 1–100 and > 100. In contrast, the percentage of black patients was significantly lower in the groups with CAC between 1–100 and > 100 when compared to the group where the CCS was 0 (zero), indicating a lower prevalence of coronary calcification in this population.

**Table 3:** Demographic and clinical characteristics and findings of coronary CT angiography in the total population and according to the coronary calcium score.

	Total (n = 211)	SCC= 0 (n = 137)	SCC 1–100 (n = 43)	SCC >100 (n = 32)	p.Value
Age	56.72 ± 9,31	54.76 ± 9.35a	59.65 ± 8.10b	61.32 ± 8,15bc	<.001^***^
Gender					.011^*^
Male, n (%)	126 (59,7)	73 (52,9)	33 (76,7)	22 (68,7)	
Female, n (%)	85 (40,3)	65 (47,1)	10 (23,3)	10 (31,3)	
Race/Ethnicity					.037^*^
Black, n (%)	156 (73,9)	109 (79.6)	29 (67.4)	18 (58.1)	
Mixed, n (%)	37 (17.5)	21 (15.3)	7 (16.3)	9 (29.0)	
Caucasian, n (%)	18 (8,5)	7 (5.1)	7 (16.3)	4(12.9)	
Risk factors					
Arterial hypertension, n (%)	158 (74,9)	99 (72.3)	35 (81.4)	24 (77.4)	.455
Diabetes mellitus, n (%)	50 (23,7)	21 (15.3)	17 (39.5)	12 (38.7)	.001^**^
Dyslipidemia, n (%)	138 (65,4)	79 (57.7)	30 (69.8)	29 (93.5)	.001^**^
Smoking, n (%)	21 (10,0)	13 (9.5)	6 (14.0)	2 (6.5)	.542
Former tabagism, n	38 (18,0)	18 (13.1)	9 (20.9)	11 (35.5)	.012^*^
Obesity, n (%)	44 (20,9)	24 (17.5)	10 (23.3)	10 (32.3)	.172
Alcoholic habits, n (%)	137 (64,9)	90 (65.7)	28 (65.1)	19 (61.3)	.898
FH of CHD n (%)	17 (8.1)	11 (8.0)	2 (4.7)	4 (12.9)	.437
SCC (Agatston units)	76.9 ± 239.4	0.0	32.7 ± 26.2	478.52 ± 4516	<.001^***^

**Notes.**

CHD Coronary heart disease CSS Coronary calcium score FH Family history

* p<.05.

** p<.01.

*** p<.001.

There was a significant statistically difference between the groups with the letters a and b; a and c; b and c.

## Discussion

In the present multi-ethnic cohort of men and women aged 30–79 years, we found coronary calcification in 42.9% of men and 23.6% of women. In addition, we found statistically significant differences in the prevalence of CAC between sexes and ethnicities/races. When compared with black and mixed-race individuals, Caucasian individuals had a significantly higher prevalence of CAC. We also observed a positive correlation between CAC and aging, and a higher prevalence of CAC in males.

Our findings are in line with a series of studies that have shown ethnic and racial differences in the prevalence of CAC. The observation that blacks have less calcification from coronary atherosclerosis than whites was first described in a large series of autopsies in 1965^14^. That study, which included 777 autopsies in Louisiana, on deceased individuals who did not die of atherosclerotic heart disease, found that the relative prevalence of calcific lesions in the 3 major coronary arteries was 20% to 75% higher in Caucasians than in blacks.

More recently, several studies have reported a lower prevalence of coronary calcification in blacks than in whites. One of the first studies in this context was conducted by Newman et al.^2^, who studied racial differences in CAC in a cohort of elderly people. CAC was measured using EBCT in 614 adults (67–99 years), with African Americans (AA) representing 23% of the study cohort. The risk-adjusted median CAC score in AA was lower than in whites for men but not for women^2^.

In the Prospective Army Coronary Calcium project, Lee et al.^3^ evaluated the relationship between CAC and race in an asymptomatic group of active-duty US Army soldiers using coronary angiotomography in 999 individuals between 40–45 years of age. The authors found that CAC was nearly twice as prevalent among whites (19%) compared with AA (10%); however, AAs had higher comorbidities, such as hypertension and glycosylated hemoglobin, compared with whites. Despite these differences, and after adjusting for risk factors, AA individuals were found to be 39% less likely to have CAC compared to whites^3^.

In the study by Kawakubo et al.^4^, the authors sought to compare four ethnic groups (whites, AA, Asians, and Hispanics) in the prevalence and extent of CAC and the rate of progression of CAC after a 7-year follow-up. After adjusting for other risk factors, AAs had a lower prevalence of CAC at baseline and follow-up and less progression than whites. Hispanics showed similar trends to AAs in having less CAC at follow-up, corresponding to less progression of CAC when compared to whites. Interestingly, this group showed no significant differences between Asians and whites. This study was one of the first to show ethnic differences in CAC progression; however, it was a cohort of mainly white individuals^4^.

The MESA was the first large prospective population-based study to compare CAC across different ethnic groups (30). This study consisted of 6814 subjects: white (38.4%), black (27.9%), Hispanic (21.9%) and Chinese (11.8%), without prior cardiovascular disease. MESA investigators found that the prevalence of CAC for men was 70.4% in whites, 52.1% in AAs, 56.5% in Hispanics, and 59.2% in Chinese. In contrast, the prevalence of CAC in women was 44.6% in whites, 36.5% in AAs, 34.9% in Hispanics, and 41.9% in Chinese. After adjusting for comorbid conditions, the relative risks of having CAC compared with whites were 0.78 in blacks, 0.85 in Hispanics, and 0.92 in Chinese. This study showed that white men and women had a higher risk of CAC compared to other ethnic groups; however, study participation varied based on ethnicity and may not have been large enough to represent these populations accurately^5^.

One of the largest studies to assess ethnic differences in CAC prevalence and severity was conducted by Budoff et al.^6^. Electron beam computed tomography (EBCT) was performed on 16,560 asymptomatic men and women [13,373 White, 1336 Asian (excluding Asian-Indian), 610 AAs, and 1256 Hispanic/Latino] aged ≥35 years. Results were generally similar to MESA. After adjusting for risk factors, compared to whites, the relative risks for men with CAC were 0.64 in AAs, 0.88 in Hispanics, and 0.66 in Asians. The relative risks for women with CAC were 1.58 for AAs, 0.84 for Hispanics, and 0.71 for Asians. Interestingly, multivariate analysis showed that AA men had the lowest CAC, while AA women were more likely to have calcification. The finding of higher CAC in AA women than in white women was different from that in MESA. However, this study was limited in its analysis, as 70% of the included population were men, and it was based on referral for cardiovascular risk assessment, which can be inherently biased^6^.

Some studies failed to show race-related differences in CAC. The Coronary Artery Risk Development in Young Adults (CARDIA) study by Bild et al.^7^ compared the prevalence of CAC in 443 AA and white men and women aged 28–40 years using EBCT. CAC was significantly different between men and women, but race was not associated with CAC in men or women before or after risk adjustment. Independent risk factors for CAC were male gender, BMI, and low-density lipoprotein. Specifically, these researchers found that CAC was present in 16.1% of black men and 11.8% of black women, compared with 17.1% of white men and 4.6% of white women. This study provided the basis for the larger MESA study by the same researchers, which found differences between ethnicities. The main reason for this difference is probably attributable to the fact that the CARDIA-based analysis was insufficient to determine differences between races and sexes^7^.

In the same context, the Dallas Heart Study evaluated the prevalence of CAC among AAs and Caucasians^8^. They evaluated CAC using EBCT in a population sample of 1289 participants. The results showed no significant difference in the prevalence of CAC between AA men and white men (37% vs 41%, respectively) or between AA women and white women (29% vs 23%, respectively). It is worth noting that this study found that most non-CAC risk factors for CHD were different between AA and white individuals and that these risk factors were associated with higher rates of CAC, which may help explain the differences in the CHD load^8^.

In turn, the study by Orimoloye et al.^9^ included 42,224 individuals [38,277 white, 1621 Asian, 977 AA and 1349 Hispanic] and revealed small overall differences in the distribution of CAC by race/ethnicity. The prevalence of CAC was higher in blacks (56.1%), followed by whites (55.8%), Hispanics (54.0%) and Asians (51.0%). According to sex stratification, 40.4% of women had CAC, compared to 63.7% of men. Black women (50.6%) had a higher prevalence of CAC compared to women of other races/ethnicities. While white (64%) and Hispanic (63.7%) men had a higher prevalence of CAC than men in the other two groups^9^.

Lastly, the study by Khurana et al.^10^ included a cohort of asymptomatic, postmenopausal women (124 AAs and 733 white) without prior CAD. Investigators found no difference between AA and white women in CCS despite higher dietary fat intake, BMI values, and prevalence of hypertension and DM among AA women. They found that CAC was predictive of cardiac risk and death at 10 years and age was a predictor of CAC increases in both ethnicities^10^.

In the study by Chia et al.,^15^ carried out in Singapore, the authors compared the prevalence of CAC in the 3 major ethnic groups (Chinese, Malaysian and Indian). Results showed no significant difference in CAC prevalence or mean score between the 3 groups (CAC prevalence: Chinese 50.4%, Malaysians 46.8% and Indians 45.7, p =.49%). The authors point out, however, that the number of Indians and Malaysians was much smaller than that of Chinese, which may restrict the power of the study to fully address the relationship between race and CAC.

In our study, we found a significantly higher prevalence of CAC in men than in women, which is in agreement with the vast majority of studies^2,5–9^. Regarding aging, our data confirm data from all studies that point to age as an independent predictor of CAC^2,5–9,12^.

The present study has a series of limitations, which we highlight: (1) In the present cohort the number of mixed-race and Caucasian patients is much smaller than that of blacks; (2) this difference may restrict the power of the study to fully address the relationship between race and CAC by gender.

## Conclusions

The present results support the notion that there are racial and ethnic differences in the prevalence of coronary calcification. The relationship between calcification and the incidence of coronary heart disease in these ethnic/racial groups needs to be further explored.

### Author statement

Study design: HM and MAAG / Data collection: CM / Writing of the manuscript: HM, PL, LC and MA.A.G / Revisions and approval of the final manuscript: All authors.

## Conflicts of interest

The authors declare no conflicts of interest and no specific funding sources for this work.
